# Using Fluorescence Recovery After Photobleaching data to uncover filament dynamics

**DOI:** 10.1371/journal.pcbi.1010573

**Published:** 2022-09-26

**Authors:** J. C. Dallon, Cécile Leduc, Christopher P. Grant, Emily J. Evans, Sandrine Etienne-Manneville, Stéphanie Portet

**Affiliations:** 1 Department of Mathematics, Brigham Young University, Provo, Utah, United States of America; 2 Institut Pasteur, Université de Paris, UMR3691 CNRS, Cell Polarity, Migration and Cancer Unit, Université de Paris, Equipe Labellisée Ligue Contre le Cancer, Paris, France; 3 Université Paris Cité, CNRS, Institut Jacques Monod, Paris, France; 4 Department of Mathematics, University of Manitoba, Winnipeg, Manitoba, Canada; Georgia Institute of Technology and Emory University, UNITED STATES

## Abstract

Fluorescence Recovery After Photobleaching (FRAP) has been extensively used to understand molecular dynamics in cells. This technique when applied to soluble, globular molecules driven by diffusion is easily interpreted and well understood. However, the classical methods of analysis cannot be applied to anisotropic structures subjected to directed transport, such as cytoskeletal filaments or elongated organelles transported along microtubule tracks. A new mathematical approach is needed to analyze FRAP data in this context and determine what information can be obtain from such experiments. To address these questions, we analyze fluorescence intensity profile curves after photobleaching of fluorescently labelled intermediate filaments anterogradely transported along microtubules. We apply the analysis to intermediate filament data to determine information about the filament motion. Our analysis consists of deriving equations for fluorescence intensity profiles and developing a mathematical model for the motion of filaments and simulating the model. Two closed forms for profile curves were derived, one for filaments of constant length and one for filaments with constant velocity, and three types of simulation were carried out. In the first type of simulation, the filaments have random velocities which are constant for the duration of the simulation. In the second type, filaments have random velocities which instantaneously change at random times. In the third type, filaments have random velocities and exhibit pausing between velocity changes. Our analysis shows: the most important distribution governing the shape of the intensity profile curves obtained from filaments is the distribution of the filament velocity. Furthermore, filament length which is constant during the experiment, had little impact on intensity profile curves. Finally, gamma distributions for the filament velocity with pauses give the best fit to asymmetric fluorescence intensity profiles of intermediate filaments observed in FRAP experiments performed in polarized migrating astrocytes. Our analysis also shows that the majority of filaments are stationary. Overall, our data give new insight into the regulation of intermediate filament dynamics during cell migration.

## Author summary

Fluorescence Recovery After Photobleaching (FRAP) is a commonly-used technique to analyze the dynamics of fluorescently-tagged proteins or structures in biology. After photochemical altering the fluorophor in a specific region, fluorescent material from the surrounding region moves into the photobleached region. Usually applied to the diffusion of soluble or membrane associate proteins, the existing models of analysis are not suitable for the elucidation of directional transport of elongated structures. Different modes of motions for the elongated structures with distributed lengths and velocities in cells are considered. First, we observe that filament lengths can be inferred from the level of noisiness. We further show the characteristics of fluorescence profile curves mainly depend on the occurrence of changes in velocities and distributions of velocities; whereas length distributions have negligible impact. Analysis of experimental data using this new framework indicates intermediate filaments transported by kinesins along microtubules in polarized migrating cells have gamma distributed velocities changing over time between pausing. Most filaments are found to be very slow or stationary with a few moving fast. This new computational approach should permit the interpretation of FRAP experimental data obtained with any directionally moving elongated structures of various lengths.

## 1 Introduction

Living organisms are in constant dynamic equilibrium. In cells, many structures appear generally static but are, in fact, formed of molecules continuously moving and exchanging with the surrounding. Fluorescence Recovery After Photobleaching (FRAP), developed in the 1970s, is an essential tool for understanding molecular dynamics within a cell [1–3]. The typical setup for a FRAP experiment involves a fluorescent probe, a microscope, and some method of photobleaching [4]. A portion of the domain where the molecule of interest is present is bleached and the recovery of fluorescence in that region is imaged over time. In order to gain quantitative information on molecular dynamics, mathematical models of diffusion are typically used. These include models of diffusion in inhomogeneous media [5], models of diffusion and binding using reaction-diffusion equations [6–8], and advection-reaction-diffusion models of active transport and diffusion [9]. In some instances, when diffusion parameters are not of interest, simpler ordinary differential equation models are used to elicit information [10]. All these models deal with analysis of soluble, generally globular, molecules. Until now there has been no analysis of FRAP data regarding the dynamics of filamentous structures.

The main example we have in mind is that of short term transport of mature intermediate filaments (IFs), one of three major fibrous structural components of the cytoskeleton. They form a filamentous network spreading throughout the cell cytoplasm and this network together with actin filaments and microtubules, plays a key role in cell polarity and migration [11]. In migrating astrocytes (the type of glial cell used in our migration experiments), the dynamics of the IF network is mainly driven by microtubule and actin mediated transport [12, 13]. Deterministic and stochastic mathematical models have been developed to describe the motion of IF driven by antagonistic molecular motors along microtubules [14, 15]. In [12], FRAP experiments of IFs were used to better understand how the IF network global dynamics are regulated in migrating and non-migrating glial cells. They showed that, during cell polarization, IF transport is mainly anterograde, oriented from the cell center to the cell periphery, and this bias was due to the inhibition of the retrograde transport of IFs by CDC42-driven polarity signaling. However, due to the high density of the IF network, it was not possible to quantify the dynamics of IFs at the single filament level. Hence, there is a need for a mathematical model to better understand collective IF transport using FRAP data.

**Fig 1:**
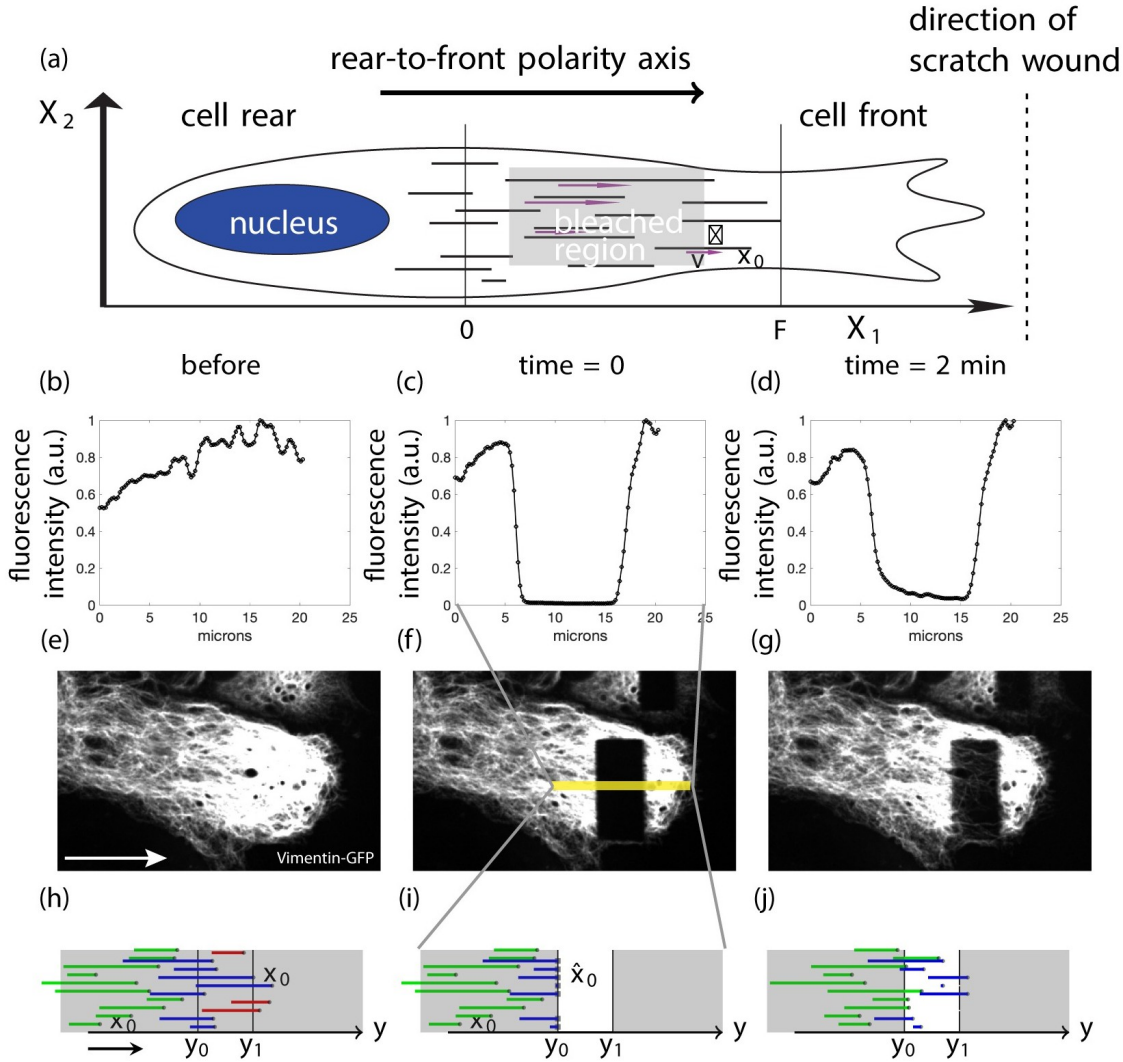
A schematic of the model and an example of a FRAP experiment. Panel (a) shows the schematic representation of a cell migrating to the right with several cytoplasmic IFs. Selected velocities are denoted with purple arrows under the filaments. The right endpoints of the IFs are denoted *x*_0_ and one is labeled with its length *ℓ* and velocity *v*. The right endpoints are distributed between 0 and *F*. Panels (b)-(g) show data from a FRAP experiment. Panels (b)-(d) show the profile curves for (e)-(g) respectively. The curves in (b)-(d) come from data in a subregion of (e)-(g); for example the yellow box in (f). Panels (e)-(g) show fluorescence images taken from a FRAP experiment performed on vimentin-EGFP expressing astrocytes, located at the edge of a wound, 1h after wounding of the monolayer. The rear-to-front polarity axis similar to the direction of migration is indicated by an arrow in (a), (e) and (h). Data are before (b) and (e), just after (c) and (f), and 2 minutes after bleaching (d) and (g). Panels (h)-(j) are blowups of the region indicated by the yellow box in (f) and show the domain and setup for the simulations. For the clarity, the vertical coordinate of each filament stays the same. The photobleached region (shown in white in (i) and (j)) is between *y*_0_ and *y*_1_. Several filaments are shown with their initial right endpoint depicted by a dot in (h)-(j). The red filaments are not relevant for our mathematical analysis and simulations since we consider only right moving filaments. Of the remaining filaments, the green ones are unbleached and the blue ones are partially bleached. In (i) the squares denoted ${\hat{x}}_{0}$ show where the new right endpoints will be after bleaching. In the mathematical analysis and simulations it is as if all the filaments to the right of *y*_0_ are bleached since we only consider right moving filaments.

There are two types of data gathered when conducting FRAP experiments. The first is a series of time measurements, called the profile curves, which show the profile of fluorescence intensity plotted along the direction of migration ($X_{1}$-direction) across the bleached region, and integrated along the perpendicular direction ($X_{2}$-direction) as depicted in Fig 1a. The second type of data is the total fluorescent intensity of the bleached region as a function of time (fluorescence intensity vs time after bleaching) and is called a fluorescence recovery curve. Since the former give more information we focus on those.

Our goal here is to determine what information can be obtained from the profile curves obtained from FRAP data. In particular, is it possible to infer the mode of filament motion (constant or variable velocity, with or without pausing), or information about filament velocity and length?

## 2 Methods

Experiments were performed in astrocytes [12] which are the major glial cells of the central nervous system. Astrocyte polarization and migration was induced by a scratch wound. We studied the IF dynamics in cells at the wound 1–2 hours after wounding, when cells are polarizing and migrate perpendicularly to the wound axis (see Fig 1a). IF are transported by molecular motors along the polarized microtubule network, which is aligned with the front-to-rear polarity axis, with the microtubule minus ends concentrated near the cell center and the plus-ends growing towards the cell’s leading edge [16]. Therefore, IF motion is also mostly parallel to the direction of migration. The bleached region is rectangular and its height is perpendicular to the protrusion, i.e. parallel to the wound (see Fig 1a). In these conditions, the fluorescence profile curve is asymmetric, due to the inhibition of the dynein-dependent retrograde transport of IFs and reflects the anterograde transport of IF dominated by kinesin motors along the polarized network of microtubule [12]. Hence in these experiments most of the transported IFs move from the center to the front of the cell and across the width of the bleached region. This allows us to reduce the problem to one dimension in the direction of the width of the bleached region ($X_{1}$ direction in Fig 1a). Furthermore, we assume the density of the filaments is uniform in the direction of the height of the bleached region ($X_{2}$ direction in Fig 1a).

The time scale of the experiments is less than 30 minutes and the fluorescence only comes back from the edges of the bleached region. Hence we assume diffusion and remodelling of the filaments due to polymerization/depolymerization, subunit exchange (which occurs on a timescale of hours), fusion or severing are negligible [12, 17]. Thus, the length of the filaments is assumed to be fixed during the observation time and the active transport of filaments moving from the cell center to the cell front is the major mechanism causing fluorescence recovery. In this model crowding effects or interactions with other filaments or organelles are not taken into consideration.

We will use mathematical modeling to determine what information can be obtained from FRAP data in the context of directional transport of elongated structures. In particular, we will focus on what characteristics of the velocity, length, and pause of filaments can be deduced from the experimental data. Due to the one directional transport, velocity and speed are synonymous in this work.

### 2.1 The mathematical model

We now mathematically frame the problem under consideration. Given a domain, $X_{1}\times X_{2}\subset R^{2}$, we place a fixed number of filaments parallel to the $X_{1}$ axis and allow these filaments to move in the positive $X_{1}$ direction (direction of the width of the bleached region) independent of the other filaments. Formally the mathematical model of motion can be stated as: let *x*(*t*) be the position of the right endpoint of the fiber at time *t*, with *v* ≥ 0 the velocity, *x*_0_ the initial position, and *ℓ* the fiber length which does not change in time. Then

$$x\left(t\right)=vt+x_{0},$$

and the left endpoint is at *x*(*t*) − *ℓ*. We consider the random variables *X*_0_, *V*, and *L*, which we assume to be independent, corresponding to *x*_0_, *v*, and *ℓ*. We define the corresponding random process *X*(*t*) = *Vt* + *X*_0_ (see Fig 1a for a depiction of the setup).

We compare our results with experiments from polarized migrating astrocytes showing asymmetric fluorescence intensity profile curves with reduced retrograde transport [12]. Thus we consider only filaments which move to the right (having non-negative velocities). Following the observations that neurofilaments, a type of IFs observed in neurons, display a stop-and-go motion [13, 18], we assume that the filaments can have a stop-and-go behavior, moving for a period of time *T*_*m*_ at a velocity *V* and then stopping for a period of time *T*_*s*_. The cycle repeats, with the motion time, velocity, and stop time for each cycle being independent of those for the other cycles.

We now consider the experimental process of FRAPing in the context of IFs (or any elongated objects transported unidirectionally). We assume the right endpoints of the filaments are uniformly distributed on the interval [0, *F*]. Suppose that *y*_0_ < *y*_1_ (the start and end of the bleach zone) and a region $\left[y_{0},y_{1}\right]\times X_{2}$ is bleached. We also assume *y*_0_ ≤ *F* to ensure that there are filaments in the bleached region at the time of bleaching. The bleaching process does not change the underlying behavior of the fibers, but the bleached portions of the fibers are no longer visible and do not contribute to the fluorescence intensity profiles (see Fig 1).

We categorize filaments into three types: unbleached filaments, partially bleached filaments, and entirely bleached filaments. Since we only use fluorescence intensity data, we only consider the unbleached portions of filaments as these are the parts of the filaments that fluoresce. For our analysis we restrict the filaments to have non-negative velocities. We need not consider filaments which do not extend to the left of *y*_0_ (i.e., for which *x*_0_ − *ℓ* > *y*_0_; red filaments in Fig 1h) and filaments whose left endpoint is to right of *y*_1_ (none of these filaments are shown in Fig 1h–1j). The rest of the partially bleached filaments (the blue filaments in Fig 1h) are defined to have a new right endpoint where the unbleached portion of the filament to the left of the bleached portion starts (marked by squares on the filaments in Fig 1i). And of course, the unbleached filaments which lie to the left of the bleached region (the green filaments in Fig 1h–1j) are considered. Mathematically we say the right endpoints of the filaments under consideration are defined in the following manner:

$${\hat{x}}_{0}=\{\begin{array}{cc} y_{0} & \text{if}\;x_{0}>y_{0}\;\text{and}\;x_{0}-\ell <y_{0}\text{,}\\ x_{0} & \text{otherwise.} \end{array}$$

### 2.2 Simulations

There are five random variables with their associated distributions in the model. The initial setup of the filaments is determined by two random variables: the initial position of the right endpoint *X*_0_ and the fiber length *L*. The movement of filaments is governed by *V* the filament velocity, *T*_*s*_ the pausing time of the filament, and *T*_*m*_ the time the filament is moving. We denote the distributions for all the random variables as follows:

-
$\mu_{X_{0}}$ governs the initial right endpoint *X*_0_ of the filaments,
- *μ*_*L*_ governs the length *L* of each filament,
- *μ*_*V*_ governs the velocity *V* of the filament during each period of motion,
- *μ*_on_ governs the duration of time each filament moves before pausing or changing velocity, and
- *μ*_off_ governs the duration of time each filament remains stationary (or pausing) before moving again.

The distribution $\mu_{X_{0}}$ that governs the initial position of the right endpoint of filaments is always a uniform distribution on the interval [0, *F*], for some *F* > 0 and *y*_0_ ≤ *F*. The distributions of the moving and pausing times, *μ*_on_ and *μ*_off_, are also always uniform and only the parameters are unknown. The distribution types and their characteristic parameters for filament length and velocity, *μ*_*L*_ and *μ*_*V*_, are also unknown. The types of distributions considered are Dirac delta (or deterministic), uniform, normal, and gamma distributions. Dirac delta distribution would reflect a case where the velocity of filament is precisely set. Uniform distribution of velocities would mean that all velocities are equiprobable, and that there is no internal control of filament velocity within the cell. For example, the velocity could be between 0 and the maximal free load velocity of kinesin motors. The Gaussian distribution would reflect the case where velocities are distributed symmetrically around an average value, suggesting that the control of filament velocity is noisy but symmetrical. The gamma distribution would reflect the fact the velocity is asymmetrically distributed with a higher contribution of slow filaments. The gamma distribution would empirically describe the asymmetric velocity distributions predicted in the transport of cargoes when friction plays an important role [19, 20], as it is the case for IF [12]. The Dirac distribution depends on one free parameter and the other three have two free parameters. The mean denoted *μ* and standard deviation denoted *σ* are defined in the standard manner for the uniform and Gaussian distributions and for the gamma distribution *μ* = *kθ* and $\sigma =\sqrt{k}\theta$ where *k* and *θ* are the free shape and scale parameters respectively. Hence the determination of the appropriate distribution and their relevant parameters to use is the primary objective of this study.

**Fig 2:**
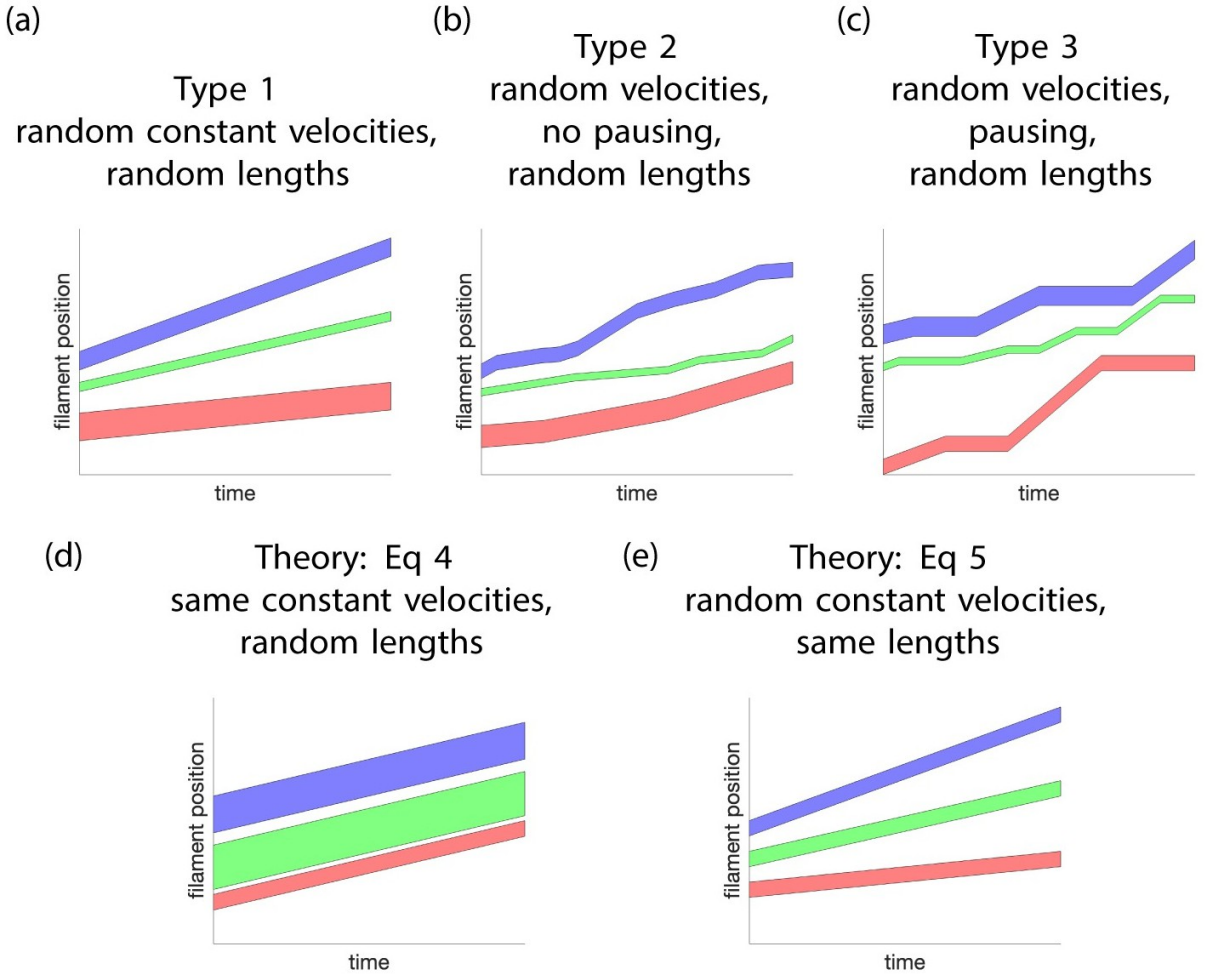
A diagram depicting the different theory and simulation types and cases solved theoretically. In each panel, kymographs of three typical filaments are shown. Panels (a)-(c) depict the three types of simulations where all the filaments have random velocities and random lengths. Panel (a) shows type 1 where the filaments have random velocities which are constant for the duration of the simulation. Panel (b) shows type 2 simulations where the filaments have random velocities which instantaneously change at random times. Finally, (c) shows type 3 simulations where filaments have random velocities and exhibit a pausing behavior between velocity changes. Panel (d) shows the particular situation/case of Type 1 simulations theoretically solved in Eq (4) where all filaments have the same fixed velocity but random (time-independent) lengths. Panel (e) the particular situation/case of type 1 simulations theoretically resolved in Eq (5) where all the filaments have the same length but different (time-independent) velocities that are randomly selected.

We numerically simulated the FRAP experiments by moving the filaments and calculating how many filaments are in the bleached region. We did this in three different ways: 1) each filament gets a different velocity determined by *μ*_*V*_ but it remains constant throughout the simulation; 2) after *T*_on_ time units have elapsed, where *T*_on_ is determined by *μ*_on_, velocities change but always come from the same distribution, *μ*_*V*_; and 3) filaments have velocities which change, again determined by *μ*_*V*_, but they stop in between velocity changes. Thus, there is a cycle for each filament of duration *T*_on_ + *T*_off_ where *T*_on_ is a *μ*_on_ distributed random variable and is the time the filament is moving (the motion is on) and *T*_off_ is a *μ*_off_ distributed random variable and is the time the filament is stationary (the motion is off). We refer to these simulations as type 1, 2, and 3 simulations. Fig 2 depicts three filaments for each type of simulation. Depending on the simulation the length of the filaments is either fixed or uniformly, normally, or gamma distributed. Similarly, the velocity is either fixed or uniformly, normally, or gamma distributed. For the velocity the normal and gamma distributions are truncated so no velocities are greater than 40 microns per minute (and for the normal distribution the velocities are all positive). For type 2 simulations the lengths of time during which velocity is fixed are uniformly distributed with a specified mean *τ*_on_. For type 3 simulations the stop and run times are uniformly distributed with specified means *τ*_off_ and *τ*_on_.

We use MATLAB to perform the simulations. This work is driven by experimental data, however the variables of interest (velocity, length, pausing, and moving time) are not observable. Hence we model the variables of interest as random variables with underlying distributions. As previously motivated we use 4 types of distributions. We estimate only the parameters (mean and variance) of these distributions. Hence we fit the simulated fluorescence intensities to FRAP data by calibrating these distributions using the MATLAB function fmincon, a nonlinear optimizer which finds the minimum of a constrained nonlinear multivariable function. The result of our fitting specifies which type of distribution to use and its relevant free parameters for each variable of interest (velocity, pausing, and moving times). We found that information about length is not encoded in the profile curves, thus, to reduce the model complexity during the fitting process, we fixed the length to be a uniformly distributed random variable with *μ* = 5.025.

### 2.3 Experiments

#### 2.3.1 Cell culture

Primary rat astrocytes were prepared as previously described [21] according to the guidelines approved by the French Ministry of Agriculture and following European standards. For scratch-induced migration assays, cells were seeded on poly-L-ornithine-precoated coverslips for immunofluorescence or 35-mm glass-bottomed culture dishes (MatTek Corporation) for videomicroscopy. Cells were grown to confluence in DMEM with 1 g/l glucose and supplemented with 10% FBS (Invitrogen), 1% penicillin–streptomycin (Thermo Fisher Scientific), and 1% amphotericin B (Thermo Fisher Scientific). On the day of the experiment, cells were scratched with a blunt-ended microinjection needle, creating a 300-*μ*m-wide wound to trigger cell migration.

#### 2.3.2 Cell transfection

Starting from a 10 cm diameter petri dish, primary astrocytes grown to confluence were trypsinized and electroporated with a Nucleofector machine (Lonza) using 5 *μ*g of vimentin-EGFP DNA. We have previously shown that EGFP-tagged vimentin co-polymerizes with the endogenous IF proteins and fluorescently labels the whole astrocytic IF network. Therefore labeling vimentin fluorescently is enough to follow the dynamics of the complete/whole IF network [12]. Medium was changed the day after transfection.

#### 2.3.3 Live-cell imaging

Nucleofected primary astrocytes were seeded on 35-mm glass-bottomed dishes and grown to confluence for 4 days. On the day before wounding, the medium was changed to a phenol red–free DMEM supplemented with 10% serum. The monolayer was wounded and cells were monitored between 1 and 2 hours after wounding, allowing them to grow a polarized protrusion [22]. Videos were acquired on a spinning-disk confocal microscope (PerkinElmer) equipped with an electron-multiplying charge-coupled device camera and either a 63×, 1.4 NA objective or a 100×, 1.4 NA objective.

## 3 Results

In this section we give the results of the mathematical theory, the three types of simulations, and the experimental data. We divide it into five main subsections: first, we explain the theoretical results; second, we consider what can be learned from the initial setup; third, we compare type 1 simulations (where the velocity for each filament is fixed for the duration of the simulation but each filament’s velocity can be different) with the theoretical results derived in Eqs (4) and (5); fourth, we compare results from type 1 simulations (where each filament can have a different but fixed velocity) with type 2 simulations (where the velocity can abruptly change to a new value during the simulation) and with type 3 simulations (where the filament pauses before changing velocity); and finally, we compare the theory and results from simulations of type 1 and type 3 with experimental data.

### 3.1 Theoretical results

Based on the filament motion model assumed in this work and the description of the experimental setup described above, we are now deriving closed forms for the profile curves. Two simplifications allow the derivation of two equations for the profile curves valid under the corresponding assumptions. First, we assume that all the filaments have the same fixed velocity (a special case of type 1 simulations where all the filaments have a fixed velocity which is the same, i.e., a Dirac delta distribution which gives all the filaments the same velocity, see Fig 2d). Thus the velocity is deterministic and no longer random. We then derive the corresponding profile curves in Eq (4). Second, we used a random non-fixed velocity and we fix an identical filament length for all filaments, see Fig 2e. This allows us to derive Eq (5).

Let *y* represent an arbitrary point in the bleached zone, i.e., *y* ∈ [*y*_0_, *y*_1_]. The probability that some part of the filament is at *y* is given by

P({X(t)>y}∩{X(t)-L<y}).

Recall we are interested only in filaments which enter the bleached region from the left. There are two types of filaments, ones which are not bleached and ones which are partially bleached. Thus we define two sets, given *y* and *t*. Let

$$\begin{array}{c} U\left(t,y\right)=\{\left(x_{0},v,\ell \right)|x_{0}+vt>y\;\text{and}\;x_{0}+vt-\ell <y\;\text{and}\;x_{0}<y_{0}\} \end{array}$$ (1)

be the set of values corresponding to filaments that are not bleached, because their right endpoints are located before the bleached region (the green filaments in Fig 1h–1j). Likewise let

$$\begin{array}{c} B\left(t,y\right)=\{\left(x_{0},v,\ell \right)|y_{0}+vt>y\;\text{and}\;y_{0}+vt-\left(\ell -\left(x_{0}-y_{0}\right)\right)<y\\ \text{and}\;x_{0}>y_{0}\;\text{and}\;x_{0}-\ell <y_{0}\} \end{array}$$ (2)

be the set of values corresponding to the non-bleached region of filaments that are only partially bleached (the blue filaments in Fig 1i–1j). We note that these two sets are disjoint so

$$P\left(\left(X_{0},V,L\right)\in U\left(t,y\right)\cup B\left(t,y\right)\right)=P\left(\left(X_{0},V,L\right)\in U\left(t,y\right)\right)+P\left(\left(X_{0},V,L\right)\in B\left(t,y\right)\right).$$

If we let *P* be the distribution of (*X*_0_, *L*, *V*), and let *E* be the corresponding expectation, then the first probability becomes

$$P\left(\left(X_{0},V,L\right)\in U\left(t,y\right)\right)=E\left(1_{U(t,y)}\right)=\int 1_{U(t,y)}\left(\omega \right)\;dP\left(\omega \right)=\int 1_{U(t,y)}\left(x_{0},v,\ell \right)\;dP\left(x_{0},v,\ell \right),$$

and we can obtain the second probability similarly. The profile curves are scaled versions of

$$\begin{array}{c} H\left(t,y\right)=\int \int \int \left(1_{U(t,y)}\left(x_{0},v,\ell \right)+1_{B(t,y)}\left(x_{0},v,\ell \right)\right)\;d\mu_{X_{0}}\left(x_{0}\right)\;d\mu_{L}\left(\ell \right)\;d\mu_{V}\left(v\right), \end{array}$$ (3)

while the fluorescence recovery curve is a scaled version of $G\left(t\right)=\int_{y_{0}}^{y_{1}}H\left(t,y\right)\;dy$.

Recall that it is assumed that filaments right endpoints are uniformly distributed; so let *X*_0_ be uniformly distributed on the interval [0, *F*] with *F* ≥ *y*_0_. Moreover, define

$$f(t,y,\ell ,v)=\int_{\left[0,F\right]}\left(1_{U(t,y)}\left(x_{0},v,\ell \right)+1_{B(t,y)}\left(x_{0},v,\ell \right)\right)\;d\mu_{X_{0}}\left(x_{0}\right).$$

In order to compute *H*(*t*, *y*) explicitly, we make one of two simplifying assumptions. First, suppose *V* = *v* with probability one (see Fig 2d). In this case it is convenient to work in traveling wave coordinates so we let *w* ≔ *y* − *vt*. In this scenario we find

$$\begin{array}{c} H\left(t,y\right)=\int f\left(t,y,\ell ,v\right)d\mu_{L}\left(\ell \right)=\\ \left\{\begin{array}{lll} \frac{1}{F}\left[\int_{-w}^{F-w}\left(w+\ell \right)d\mu_{L}\left(\ell \right)+\int_{F-w}^{\infty }Fd\mu_{L}\left(\ell \right)\right] & \text{if}\;w\leq 0, & \left(4\text{a}\right)\\ \frac{1}{F}\left[\int_{0}^{F-w}\ell d\mu_{L}\left(\ell \right)+\int_{F-w}^{\infty }\left(F-w\right)d\mu_{L}\left(\ell \right)\right] & \text{if}\;0<w\leq y_{0}, & \left(4\text{b}\right)\\ 0 & \text{if}\;y_{0}<w. & \left(4\text{c}\right) \end{array}\right. \end{array}$$

Note that *H* depends on (*t*, *y*) through the traveling wave coordinate *w*.

Second, suppose instead that all filaments have the same length *ℓ* with probability one (see Fig 2e). In this case the only genuine random variables are *X*_0_ and *V*. Then we have

$$\begin{array}{c} H\left(t,y\right)=\{\begin{array}{cc} \frac{1}{F}[\int_{\frac{y-F+\ell }{t}}^{\frac{y+\ell }{t}}\ell \;d\mu_{V}\left(v\right)+\int_{\frac{y-y_{0}}{t}}^{\frac{y-F+\ell }{t}}\left(F-y+vt\right)\;d\mu_{V}\left(v\right)\\ +\int_{\frac{y}{t}}^{\frac{y+\ell }{t}}\left(y-vt\right)\;d\mu_{V}\left(v\right)] & \text{if}\;\ell \leq y_{0}\;\text{and}\;F\leq y_{0}+\ell \text{,}\\ \frac{1}{F}[\int_{\frac{y-y_{0}}{t}}^{\frac{y+\ell }{t}}\ell \;d\mu_{V}\left(v\right)+\int_{\frac{y}{t}}^{\frac{y+\ell }{t}}\left(y-vt\right)\;d\mu_{V}\left(v\right)] & \text{if}\;\ell \leq y_{0}\;\text{and}\;F>y_{0}+\ell \text{,}\\ \frac{1}{F}[\int_{\frac{y-F+\ell }{t}}^{\frac{y+\ell }{t}}\left(y-vt+\ell \right)\;d\mu_{V}\left(v\right)+\int_{\frac{y-y_{0}}{t}}^{\frac{y-F+\ell }{t}}F\;d\mu_{V}\left(v\right)\\ +\int_{\frac{y-y_{0}}{t}}^{\frac{y}{t}}\left(vt-y\right)\;d\mu_{V}\left(v\right)] & \text{if}\;\ell >y_{0}\;\text{and}\;F\leq y_{0}+\ell \text{,}\\ \frac{1}{F}[\int_{\frac{y-y_{0}}{t}}^{\frac{y+\ell }{t}}\left(y-vt+\ell \right)\;d\mu_{V}\left(v\right)+\int_{\frac{y-y_{0}}{t}}^{\frac{y}{t}}\left(vt-y\right)\;d\mu_{V}\left(v\right)] & \text{if}\;\ell >y_{0}\;\text{and}\;F>y_{0}+\ell \text{.} \end{array} \end{array}$$ (5)

Hence Eq (4) represents the density of fluorescent filaments at time *t* and location *y*, or the theoretical profile curves, when the velocity is fixed, and Eq (5) represents the density of the fluorescent filaments (or profile curves) when the length is fixed. The details of these calculations are found in S1 Appendix.

### 3.2 Initial setup

**Fig 3:**
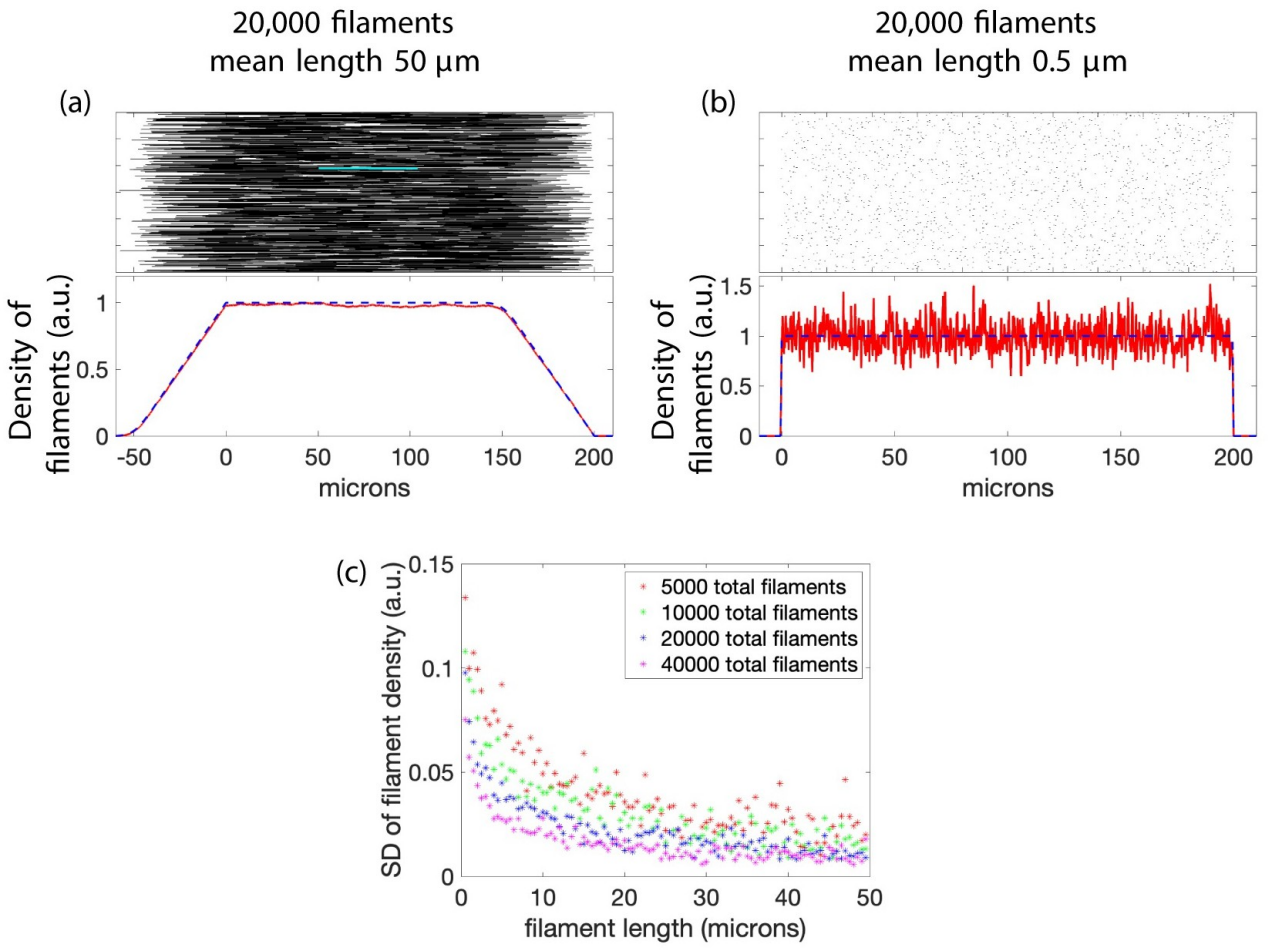
Impact of filament length on the density of filaments. This figure shows how filament length affects density measurements of the initial setup (before bleaching, all filaments are fluorescent). The mean length of filaments in (a) is 50 microns and in (b) 0.5 microns. One filament is highlighted in cyan in (a). The red lines show the density of filaments computed from the data shown above (calculated using bin sizes of 0.1 in (a) and 0.09 in (b)), normalized using the maximum bin value (a) and average non-zero bin value (b), and the dashed blue line shows the theoretical density of filaments using Eq (4), where *y*_0_ is set to be greater than *F* (this is the one exception to the assumption that *y*_0_ ≤ *F*), thus there is no bleached region in the panels. The filaments’ right endpoints are uniformly distributed on [0, 200] and the filaments have lengths which come from a Gaussian distribution with standard deviation 5 in (a) and 0.05 in (b). Eq (4a) is for *w*-values less than 0, where the density is lower since the right endpoints of filaments are not initially placed to the left of 0 (see panel (a)). For panel (a), if *F* − *w* > 60 (the mean length plus 2 standard deviations), the *w* coordinate is far enough to the left of *F* = 200 that the boundary effects (due to placement of the right endpoints) do not affect density. If *y*_0_ is in the plateau region, the front of the traveling wave will be sharp. Different length distributions show the same qualitative features. Panel (c) shows the standard deviation of the filament density for different lengths of filaments and for four different values of total number of filaments. The simulations in (c) have filament lengths which are Gaussian distributions with varying length and the standard deviation is one tenth the length. The standard deviation is taken only for data on the plateau.

As will be shown in Section 3.3, the mathematical theory indicates that data from FRAP experiments reveals little information about filament length distributions, however some information can be obtained. By knowing how the density of the filaments changes in time and space some limited information about filament length can be deduced. In order to explain this we consider the initial setup for the system and distinguish between the initial distribution of the right endpoints of filaments and the distribution of filament densities. The first is independent of filament length *ℓ* and the second is not. For our mathematical setup we consider the filaments where the right endpoints are uniformly distributed in the interval [0, *F*] (see Fig 1a). The normalized density of filaments as a function of space depends on the length of filaments; two examples are shown in Fig 3. The normalized density of filaments as a function of space will increase until it reaches 1 and then remain constant until some point before *F* where it will decrease to 0 at *F* and remain 0 from there on. The regions of increase and decrease are determined by the length distribution of the filaments. These types of regions may be found near the cell membrane. In panel (a) of Fig 3 the filament lengths have a larger mean (50, ±5*μ*m (SD) from a Gaussian distribution) and thus have a gentle slope. In contrast, in panel (b) of Fig 3 the lengths have a smaller mean and standard deviation (0.5, ±0.05*μ*m (SD) from a Gaussian distribution), resulting in a much sharper transition. In addition, if the filaments are long, the density measured from data will be smoother (less variability, with fewer filaments) but if the filaments are short, the measured density will be noisy (more variability which will require more filament measurements to smooth the density signal) as seen in panel (b).

To summarize, filaments with a short average length will give density measurements with greater variability and spatial derivatives. Measurements near the cell membrane may give an idea of average filament length due to the boundary.

### 3.3 Type 1 simulations and theory

In type 1 simulations the velocity for each filament does not change with time (see Fig 2). First we consider a special case of this, namely when the velocity is the same for all the filaments and the filament length, which does not change during the course of the experiment (here we do not model filament assembly or disassembly), is random. Then we consider fixed lengths and random (but constant) velocities. Finally we consider both random velocities and random lengths.

#### 3.3.1 All filaments have the same constant velocity

**Fig 4:**
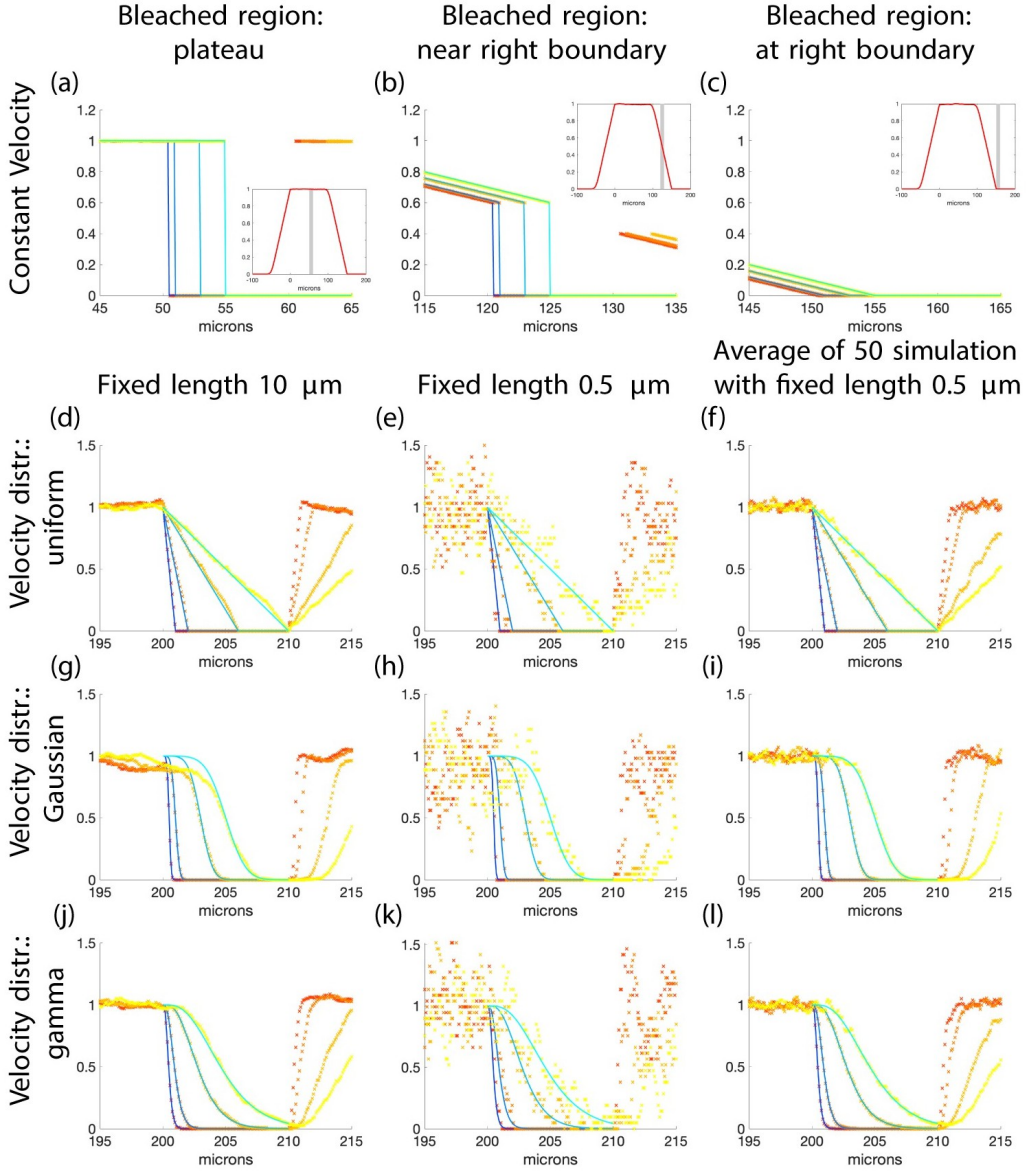
Impact of velocity distributions and filament length on FRAP intensity profile curves from the theory and type 1 simulations. The solid curves in (a)-(c) are plots of a scaled version of Eq (4) and in (d)-(l) they are a scaled version of Eq (5) such that ‖*f*‖_∞_ = 1. The x’s are simulated results. The mean velocity for all simulations is 1 micron per minute and for (g)-(l) the standard deviation of the velocity is 0.25. In (a)-(c) the velocity is fixed and the insets show the simulated profile curves for the entire domain before bleaching and the region to be bleached is shown in grey. The bleached region is 50–60, 120–130, and 150–160 respectively. In (d)-(f) the velocity is uniformly distributed, (g)-(i) have Gaussian distributions, and (j)-(l) have gamma distributions. Panels (d), (g), and (j) have filaments with length 10 microns; (e), (h), and (k) have filaments with length 0.5 microns; and (f), (i) and (l) shows the average of 50 simulations (each a different realization) with filament length 0.5 microns. In these simulations, each filament has a velocity which does not change for the duration of the simulation. Each simulation in (a)-(c) has 200,000 filaments and in (d)-(l) 20,000. The right endpoints are uniformly distributed in (a)-(c) from 0 to 100 and in (d)-(l) from 0 to 470. The bleached region in (d)-(l) is from 200 to 210. The curves and x’s represent times 0.5 (blue and red), 1 (light blue and orange), 3 (lighter blue and light orange), and 5 minutes (cyan and yellow). The *y* axis is fluorescence intensity (a.u.) in all panels. **Length**: Row one—Gaussian, *μ* = 50, *σ* = 5 microns. Rows 2,3, and 4—fixed.

For fixed velocity, Eq (4) shows that the profile curves are traveling waves since they depend only on the traveling wave coordinate *w* = *y* − *vt*. In other words, the shape of the profile curve does not change but rather is shifted to the right over time. When the bleached region is where the filament density is constant (see Fig 3a and 3b) the profile of the traveling wave will not give much information about the length distribution. Recall that we assume the right endpoints of the filaments are uniformly distributed in the region [0, *F*]. Because we are placing the right endpoints in [0, *F*], the density of filaments ramps up from zero to a constant, remains constant from zero to some value below *F*, and then ramps down to zero at *F* (see Fig 3a and 3b). Thus Eq (4a) relates to the region left of the right endpoint placement (for instance Fig 3a left of 0) where the density is zero or ramping up. Eq (4b) describes the wave in the rest of the domain up to the bleached region, and Eq (4c) describes the wave to the right of the left edge of the bleached region. We consider only positive velocities and values in the bleached region, the main variations of concern are the transition from the non-bleached region to the bleached region, that is values near *y*_0_. For values of *w* such that *F* − *w* > *u* where *u* is a value such that $\int_{0}^{u}d\mu_{L}\approx 1$, the first integral in Eq (4b) dominates and is the mean filament length. In this scenario, the wave front is far enough away from *F* so the profile curve is constant (the boundary effects are negligible—see Figs 3 and 4). Thus the profile curve will be a wave which jumps down from a constant value (determined by the first integral in Eq (4b)) to zero (when Eq (4c) is used) and the only information about the length distribution that can be determined is the integral condition given above which says something about the length of the interval “containing” most of the density of *L*.

As an example see the first row of Fig 4. In Fig 4a, curves of a scaled version of Eq (4) are shown with data from simulations where the bleached region is in the region where the filament density is uniformly distributed (not just the right endpoints). The filament density is shown for the full domain before bleaching in the inset. The region to be bleached, between *y*_0_ = 50 and *y*_1_ = 60, is shown as grey. The profile curves are constant with a jump at the transition to the bleached region. In Fig 4b the bleached region is in a region near the right end of the interval where the filaments are located. In this region the filament density is not uniform and the profile curves are not constant before they drop down to zero. In Fig 4c the bleached region is at the right end of the region where the filaments are initially located *y*_0_ = *F* = 150. The profile curves here are continuous and have no abrupt transition to zero. Regardless of the length distribution, if the bleached zone is in the region of the domain where the filament density is constant, the profile curves are constant with an abrupt change to zero.

To summarize, typically, if the filament velocities are constant the wave profile will have a front at *y*_0_ which moves forward into the bleached zone without changing shape and there is almost no information about the length distribution. It may be possible to learn something about the filament lengths if the bleach region is near the cell membrane where the filament density may not be constant due to the boundary imposed by the membrane.

The fluorescence intensity profiles obtained experimentally are not traveling waves (see Fig 1c and 1d). They have an abrupt transition at the time of bleaching from the fluorescent region to the bleached region, but as time evolves the transition becomes smoother and less abrupt. In order to explain the experimental data, we explored the effects of random filament velocity on the curves. From now on, the bleached region will always be in the “plateau” region where both the right endpoints of filaments and the filament density are uniformly distributed to avoid boundary effect in the theoretical and simulated results. Since we only consider filaments moving to the cell periphery, the right side of the profile curves and simulations do not give any additional information.

The rest of section 3.3 will show a primary result of this work—that **the fluorescence intensity profile is affected only by filament velocity, and not by filament length**.

#### 3.3.2 Filaments with random velocity and fixed length

The filament velocity distribution affects the fluorescence intensity profiles profoundly. The equation derived for fluorescent filament density with random velocities and fixed length filaments (Eq (5)) is compared to numerical simulations of type 1 in Fig 4d–4l. When the velocity is uniformly distributed the profiles are piecewise linear with the slope changing with time. For Gaussian distributed velocities the profiles are smooth curves with more abrupt transitions than the uniformly distributed velocities. The profile curves are also smooth curves in the case of gamma distributed velocity but the transitions are not as abrupt as when the Gaussian distribution is used.

We then turned our attention to how changes in the fixed length parameter affects the results of type 1 simulations. It is clear from Fig 4d–4l that as the length of the filaments increases the profiles from simulations approach the theoretical curve which is determined by the velocity distribution. If the length is small, there is more variation in the simulated results due to the random nature of the simulations but that variation can be averaged out giving profiles which are similar to those with filaments of longer lengths.

**Fig 5:**
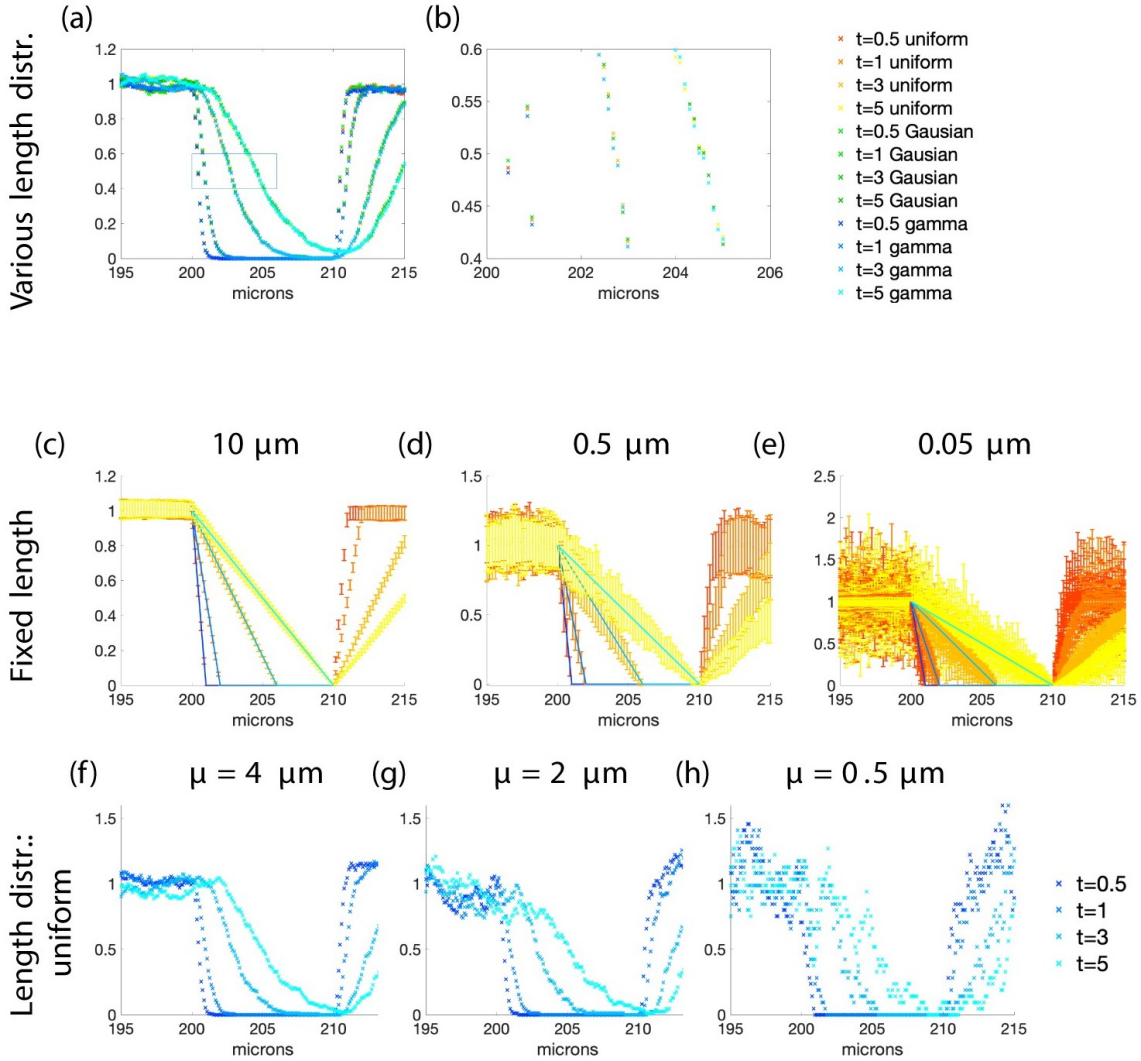
Impact of length distributions on FRAP intensity profile curve data (type 1). This figure compares the simulated results with random velocity and different length distributions. In panel (a) the type of length distribution changes, in panels (c)-(e) the length changes, and in (f)-(h) the mean length changes. Panel (b) is a blowup of the boxed region in panel (a). In panel (a) the average length for all distributions is 10 microns and for the Gaussian and gamma distributions the standard deviation is 0.2. In panels (c)-(e) the solid curves are plots of scaled Eq (5) such that ‖*f*‖_∞_ = 1 and the error bars are type 1 simulated results. The length of the error line is twice the standard deviation of the 50 realizations centered at the average of the realizations. The curves and error bars in (c)-(e) are profiles at times 0.5 (blue and red), 1 (light blue and orange), 3 (lighter blue and light orange), and 5 minutes (cyan and yellow). The *y* axis is fluorescence intensity (a.u.) in all panels. **Length**: (a)—varied distributions, (c)-(e)—fixed, (f)-(h)—uniform. **Velocity**: (a),(b), (f)-(h) gamma *μ* = 1, *σ* = 0.25; (c)-(e)—uniform on interval [0, 2].

Fig 5c–5e, shows how the standard deviation of type 1 simulations vary with the filament lengths. In these simulations (similar to those shown in Fig 4) the averages of 50 simulations are plotted with error bars indicating the standard deviation for simulations with filaments of varying lengths and with uniformly distributed velocities. Clearly the variation increases as the filament length decreases. Depending on the quality of the data it may be able to surmise length information based on the noise in the data.

#### 3.3.3 Filaments with random velocity and random length

Next, we allowed the length to vary according to different distributions with the same mean while keeping the velocity distribution fixed as a gamma distribution. We do not have a theoretical curve to compare with these type 1 simulations because both velocity and length are random. Fig 5a, curves obtained with the three different distributions (uniform, Gaussian and gamma with the same mean length) for lengths are almost superimposed. In panels (f)-(h) the length distribution was left the same (a uniform distribution) but the mean length was altered from 0.5, 2, 4 microns. Again when the filaments have shorter lengths the data is noisier but still follows the same basic curve (determined by the velocity distribution).

### 3.4 Comparing type 1, 2, and 3 simulations

Here we compare results from type 1, 2, and 3 simulations with fixed filament length and various random velocities. Recall we simulated the velocity in three ways: type 1 each filament gets a different velocity but it remains constant throughout the simulation, type 2 velocities change after *T*_on_ time units have elapsed but always come from the same distribution, and type 3 filaments have velocities which change after *T*_on_ time units but then they stop in between velocity changes for *T*_off_ time units.

**Fig 6:**
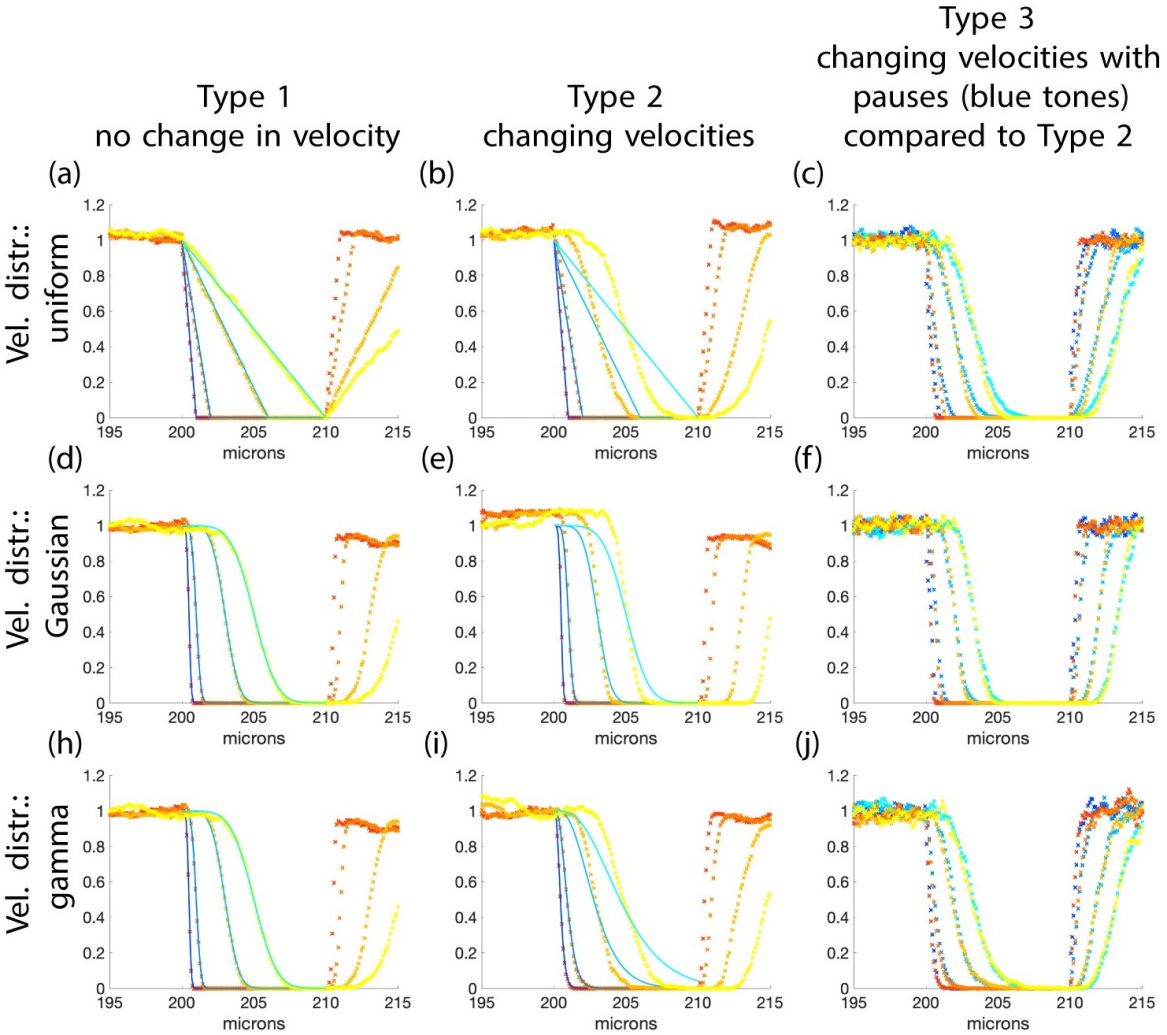
Impact of velocity changes and pauses on intensity profile curves. This figure shows how simulations differ between type 1, 2, and 3. The solid curves are plots of scaled versions of Eq (5) such that ‖*f*‖_∞_ = 1 and the x’s are simulated results. The curves and x’s represent times 0.5 (blue and red), 1 (light blue and orange), 3 (lighter blue and light orange), and 5 minutes (cyan and yellow). Panels (a), (d), and (h) show type 1 simulations with x’s and theoretical curves from Eq (5) (panels are the same as (d), (g), and (j) of Fig 4). Panels (b), (e), and (i) show type 2 simulations with x’s where the filaments change velocity after a random time chosen from a uniform distribution with mean 1 minute (the theoretical curves are for comparison with column 1). In (c), (f), and (j) the blue x’s show results from type 3 simulations where the stop time comes from a uniform distribution with mean 0.5 (on average $\frac{2}{3}$ of the initial filaments are moving, $\frac{\tau_{\text{on}}}{\tau_{\text{on}}+\tau_{\text{off}}}=\frac{2}{3}$) and the yellow x’s show results from type 2 for comparison (with the mean velocity $\frac{2}{3}$ of the comparable type 3 simulation). In (a)-(c) the velocity is uniformly distributed, in (d)-(f) it has a Gaussian distribution, and in (h)-(j) it has a gamma distribution. All simulations have filaments with length 10 microns. The other parameters are the same as in Fig 4. The *y* axis is fluorescence intensity (a.u.) in all panels. **Length**: All—fixed.

Simulations of type 1 match the results from Eq (5) (Fig 6a, 6d and 6h). For type 2 simulations, the initial part of the wave front is slower (the filaments with larger velocities on average do not maintain the large velocity and thus they do not move as far into the bleached region as before) but the back of the wave front is faster (on average the filaments with slow velocities do not remain slow). The overall effect of the change is to make the transition from the bleached region to the unbleached region more abrupt than before (Fig 6b, 6e and 6i). This has the largest effect in the case of the uniform distribution where the transition is linear for type 1 but nonlinear and sharper for type 2 (Fig 6b). For type 3 simulations, filaments paused for a period of time before changing velocity. The pausing time follows a uniform distribution with mean 0.5 minutes. The resulting profile curves are similar to the profiles of simulations of type 2 (without the pausing) except the velocity of the profiles is multiplied by the fraction of filaments that are moving, $\frac{\tau_{\text{on}}}{\tau_{\text{on}}+\tau_{\text{off}}}$, where *τ*_on_ is the mean time the filaments have a fixed velocity and *τ*_off_ is the mean time the filaments are paused before changing velocity (Fig 6c, 6f and 6j). The profiles are somewhat advanced at the front end and somewhat delayed at the back end. The pausing seems to slightly ameliorate the sharpening of the wave caused by the velocity changes. Type 2 and type 3 simulations give similar results. **When filament velocity is a random variable, the simulated profile curves are not traveling waves and the abrupt change at the time of bleaching is smoothed out as time advances as is seen in the experiments**.

### 3.5 Fitting theory and simulations to the data

We have a theoretical formula (Eq 5), and three types of simulations to compare to experimental data. We do not compare type 2 simulations to the data for two reasons. First, the results from the last section show that simulations of type 2 and type 3 are similar. Second, for long *τ*_on_ (i.e., longer than the duration of the experiment) type 2 and type 3 simulations are the same. We now optimize the parameters in the theory, type 1, and type 3 simulations to fit the experimental data.

We fit thirteen data sets from five different experiments. All the data was from polarized migrating astrocytes showing asymmetric profiles of fluorescence recovery indicative of the polarization of the microtubule driven transport of IFs [12].

**Fig 7:**
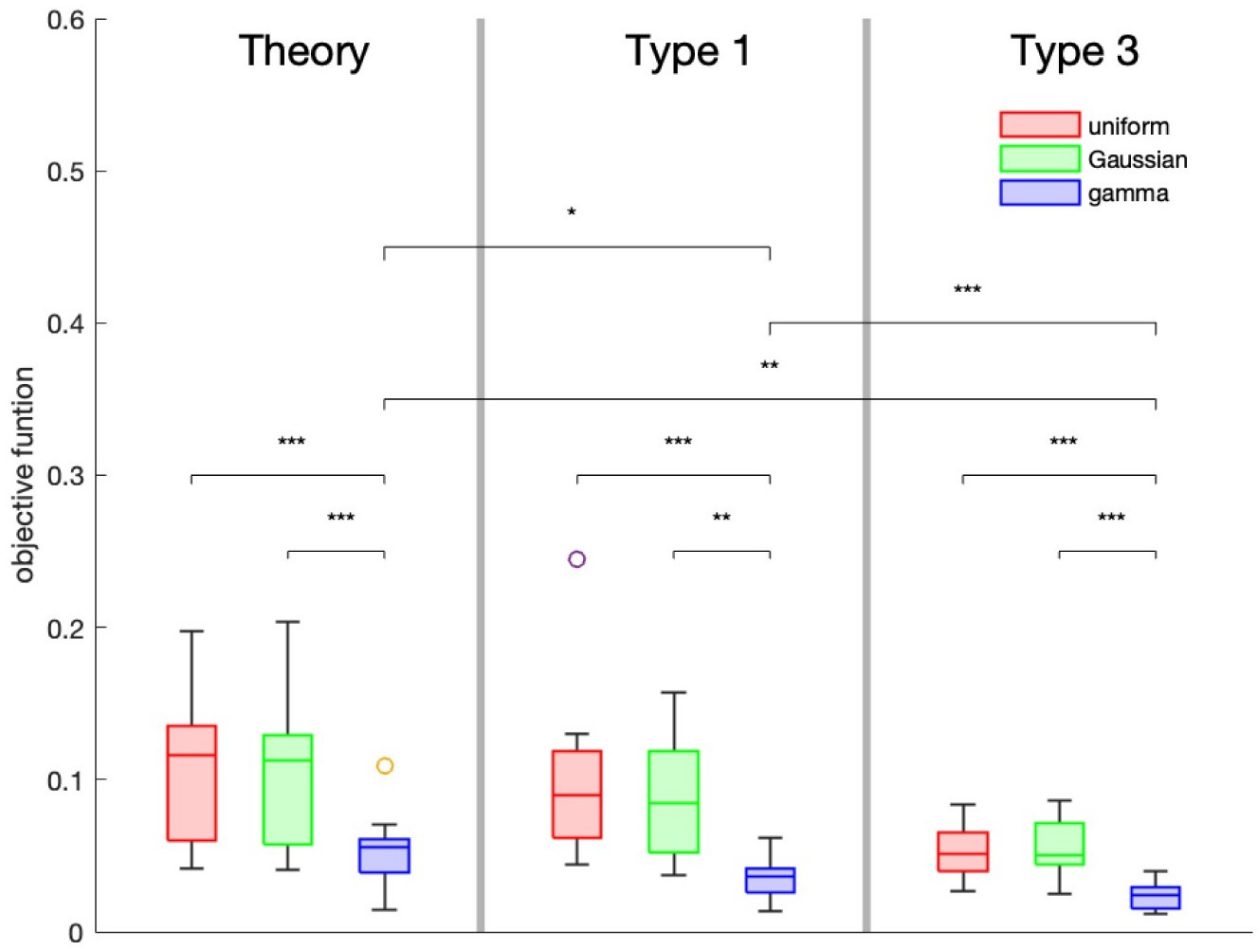
Comparison of how well the different types of simulations fit the experimental data. The results of the optimization are shown. The box color indicates velocity distribution used: red—uniform, green—Gaussian, and purple—gamma. The line in the box gives the average of the objective function, E for the best fit (out of 30) for each of the 13 data sets, the width of the box shows the upper and lower quartile, and the whisker lengths are about ±2.7*σ* where *σ* is the standard deviation. The circles are outliers. Using the Wilcoxon signed rank test: * for *p* < 0.022;** for *p* < 0.0012; *** for *p* < 0.0005. All possible pair combinations are statistically significant except the 6 possible pairings of Theory uniform, Theory Gaussian, Type 1 Uniform, and Type 1 Gaussian. In addition the difference between Theory gamma vs Type 3 uniform and Theory gamma vs Type 3 Gaussian are not statistically significant. The theory uses Eq (5). **Length**: Theory—fixed with *μ* = 10, Type 1 and 3—uniform with *μ* = 5.025 microns.

In all the simulations, filament length is uniformly distributed on the interval [.05, 10] with mean 5.025 microns. For computational convenience we shifted the distribution away from 0. For the theory and type 1 simulations we optimized over the average velocity, *μ*, for the uniform distribution, the average velocity, *μ*, and standard deviation, *σ*, for the Gaussian and the gamma distributions which is parameterized by the shape parameter *k* and the scale parameter *θ*. (Recall the mean *μ* = *kθ* and the standard deviation $\sigma =\sqrt{k}\theta$.) Both the Gaussian and the gamma distributions are truncated so the velocities do not exceed 40 microns per minute (and are not negative for the Gaussian). For type 3 simulations the mean off, *τ*_off_, and mean on time, *τ*_on_, are also free parameters. Thus type 1 simulations have one or two free parameters and type 3 simulations have three to five free parameters. The type 1 and type 3 simulations are stochastic processes so we optimized 30 realizations for each data set. The objective function minimized was

$$E=\sum_{i=1}^{N}\sum_{j=1}^{M}\frac{|s\left(t_{i},x_{j}\right)-d\left(t_{i},x_{j}\right)|}{MN}$$

where *s*(*t*_*i*_, *x*_*j*_) is the fluorescence intensity of the simulated data, *d*(*t*_*i*_, *x*_*j*_) is the experimental data, *N* is the number of time points, and *M* is the number of spatial points. Fig 7 shows the average of the minimum of the objective function E (best fit simulation) for each of the data sets. Our model comparisons are only based on how well the model data fits the experimental data. As the models considered are not nested, statistical tests are not applicable for comparison.

**Fig 8:**
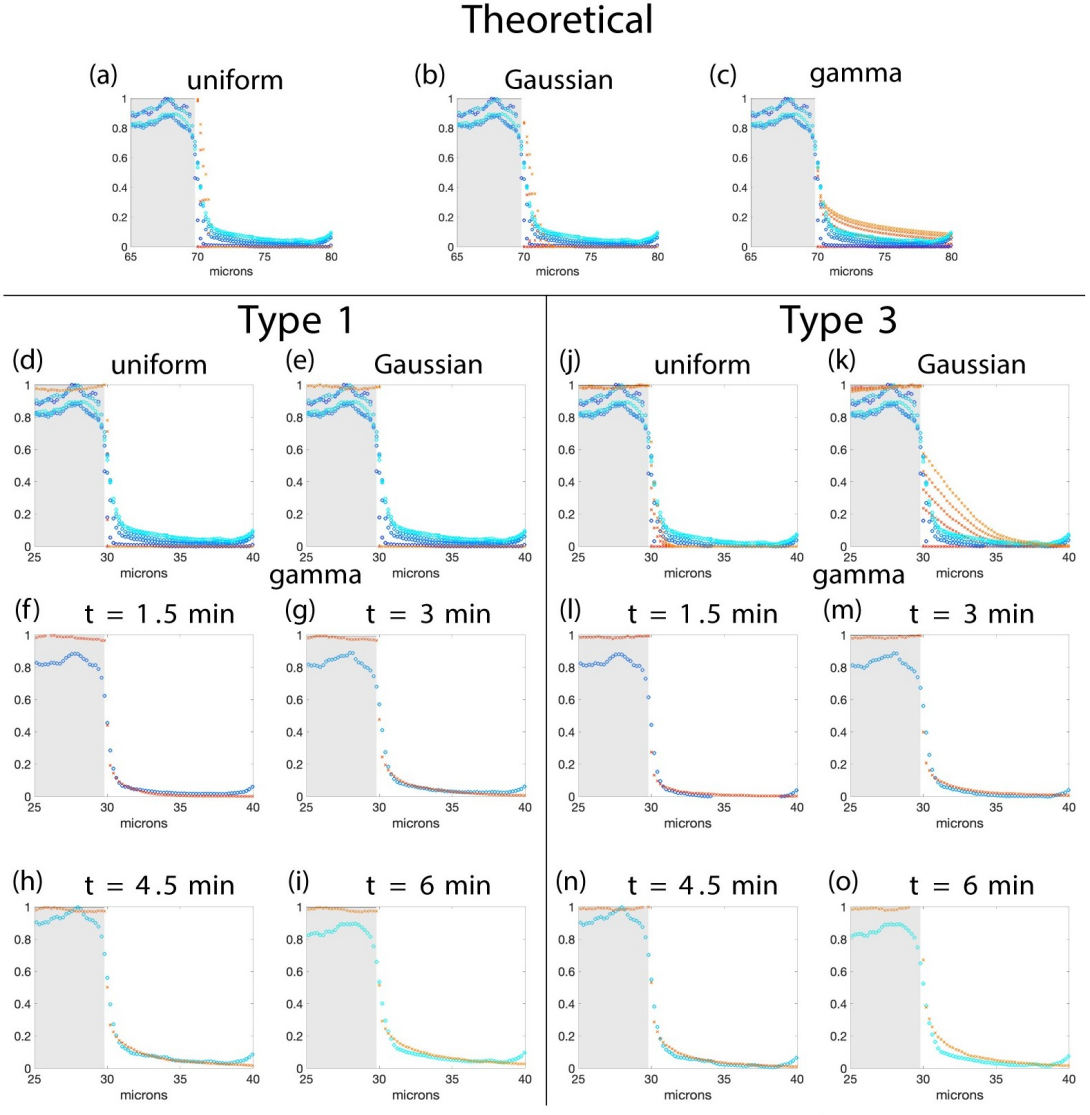
Fitting simulations to the experimental data. This figure compares the simulated results (red shades) with experimental data (blue shades) for one data set. The figure has been separated into three regions showing results from the theoretical model, type 1 simulations, and type 3 simulations. Panels (a)-(e), (j), and (k) show all the time data together, whereas the rest show the time data in individual panels. When optimizing only the data in the unshaded area is used. The minimum value of the objective function is 0.0511, 0.0525, and 0.0139 for type 1 and for type 3 0.0415, 0.0404, and 0.0131 for uniform, Gaussian, and gamma distributed velocity respectively. The *y* axis is fluorescence intensity (a.u.) in all panels. **Length**: All—uniform with mean 5.025 microns.

Although, both type 1 and type 3 simulations fit the data well, the best fits come from type 3 simulations as can be seen, for one data set, in Fig 8. Furthermore, in all cases we successfully simulated most of the fluorescence curves using a truncated gamma distribution for the velocities. This is not surprising since the videos of FRAP experiments clearly show a strong disparity in the filament speeds, with only a few filaments moving very fast and a large majority moving very slowly (see videos [12]). The gamma distribution is the only asymmetric distribution considered thus allowing for a fat tail.

**Fig 9:**
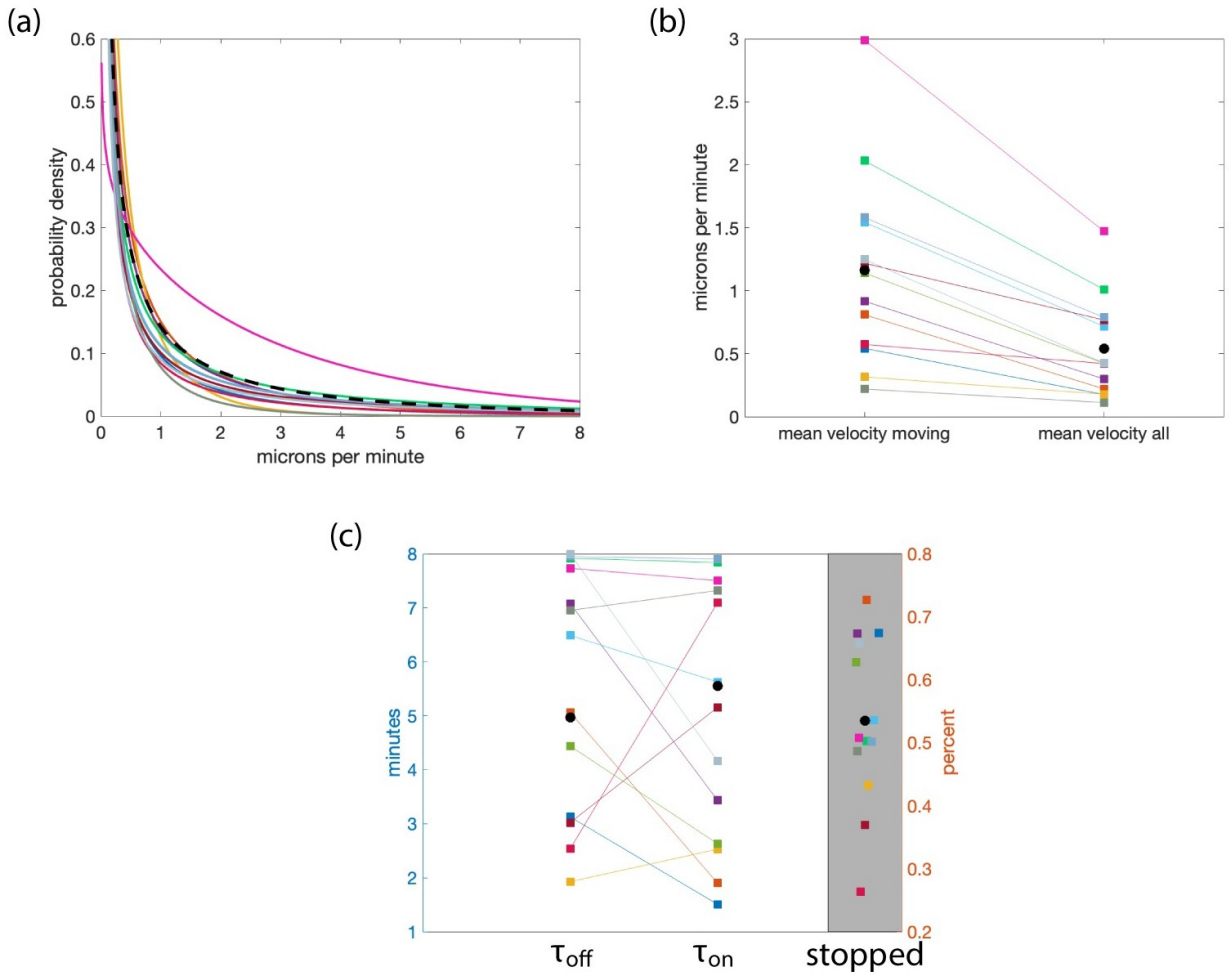
Information on filament dynamics approximated from 13 experimental data sets. Panel (a) shows the non-truncated velocity distributions as predicted by type 3 simulations (giving the minimum cost function of the 30 realizations) for each data set in a different color. All simulations used a truncated gamma distribution for the velocities. The black dotted line shows the gamma distribution with parameters which are the average of the parameters for the 13 other curves. Panel (b) shows the mean velocities. The squares to the left are velocities for moving filaments *v*_on_ and the squares to the right are velocities for all the filaments i.e., $v=v_{\text{on}}\frac{\tau_{\text{on}}}{\tau_{\text{on}}+\tau_{\text{off}}}$. Panel (c) shows the mean on and off times predicted by type 3 simulations for the 13 data sets considered. The squares to the left are the mean off times, *τ*_off_, and the squares to the right are the mean on times, *τ*_on_. The black circles are the averages of mean off and on times over the 13 data sets. The grey region shows the percent of filaments which are stopped (the horizontal coordinate is randomly perturbed for viewing purposes). Both on and off times are uniformly distributed. The lines connect values from the same data sets. The colors indicate the same data set. **Length**: All—uniform with mean 5.025 microns.

Using the scenario (type 3 simulations with gamma distributed velocities) representing the best experimental intensity profile curves, information about the filament dynamics can be extracted from the 13 data sets considered. Fig 9a summarizes the filament velocity distributions, the mean velocities of all filaments and only moving filaments (Fig 9b), the filament mean off and on times, and the percentage of stationary filaments (Fig 9c). The mean velocities of moving filaments are found to range from 0.004 to 0.05 microns per second (with an average over the 13 data sets of 0.0194) and the average velocities of all the filaments (including moving and stopped filaments) and range from 0.003 to 0.025 microns per second (with an average of 0.0108). On average the percentage of stopped fibers at steady state is $\frac{\tau_{\text{off}}}{\tau_{\text{on}}+\tau_{\text{off}}}$. Thus the data shows that in nine of the 13 data sets over half the filaments at any time are stationary (Fig 9c).

## 4 Discussion

The work here builds a theoretical framework to quantitatively analyse the directed transport of anisotropic structures and allows the reconstruction of fluorescent profile curves generated by FRAP experiments. Here the focus is on IFs but the work is more generally applicable to all kind of anisotropic structures, such as mitochondria which are also actively transported along microtubules [23]. We show that data from FRAP experiments on IF, namely fluorescence intensity profile curves, reveal important information for determining the velocity of the filaments including mean velocity and shape of the distribution. In fact, the most important distribution governing the shape of the intensity profile curves is the distribution of the filament velocity, *μ*_*V*_. Profile curves which are observed to be traveling waves would suggest that all filaments are moving with the same constant velocity. On the contrary, profile curves which are piecewise linear with the slope changing in time suggest that the filament velocity is constant in time and uniformly distributed. Profile curves which are sigmoidal indicate velocities are normally or gamma distributed. Finally, we found that the filament length distribution has no impact on the global dynamics of filaments.

Our results show that for polarized migrating astrocytes with reduced retrograde transport, a gamma distribution for the velocity of the filaments best matches the data. Allowing the filaments to pause and restart with new velocities, gives the best experimental fit. In fact, in most of the data sets over half the filaments are stationary and most of the moving filaments have a small velocity. However, some filaments have a large velocity as indicated by the long tails in the velocity probability densities in Fig 9a and the long tails in the profile curves in Fig 8. This is consistent with IF transport by one directional motor molecules with friction and with IF experiments where velocities were described to be non Gaussian with a high propensity of slow filaments in previous work [19, 20, 24]. Moreover, average velocities extracted from the fit of the profile curves with gamma distributions are consistent with values published in the literature [25], but lower than the range described for the transport of isolated small filaments called squiggles [25, 26] and longer filaments measured in [17]. The distributions are consistent with filaments having a large range of velocities which is seen experimentally [25]. All of these velocities are lower than the maximum velocity of an individual kinesin-1 molecule of 0.668 microns per second [27] but which depends on the concentration of ATP and can be as low as 0.045 microns per second for low concentrations. Additionally, increasing the load on the kinesin molecule will decrease the velocity [27]. Long pauses have also been observed to last up to 25–80 percent of the time [25]. This matches our model predictions that at any one time fewer than half of the filaments are moving and the average percentage of time the filaments are paused ranges from 25 to 73 (with an average of 53.5).

The very slow speeds are not surprising because there are a lot of sources of friction for the transport of IF mainly driven by kinesin motors: interaction with other organelles, crosslinking proteins between microtubules and intermediate filaments such as plectin, or dyneins, although dynein activity has been shown to be inhibited [12]. Maintaining a network of filaments that is constantly being restructured requires a delicate balance where a portion of the network is stable, a portion is being dismantled, and a portion is being constructed.

Our results in the case of one directional transport also predict that other types of profile curves can be recovered with velocities which are not gamma distributed. These predictions could be used in other types of experiments investigating different cell conditions not described in [12]. For example, when the friction in the system is reduced, we expect the velocity distributions to be more symmetrically distributed (Gaussian) because only one motor is involved. This could be achieved by reducing the cross-linking of the filaments. Two possible methods would be to inhibit plectin, or completely inhibit dynein. Uniform and Dirac velocity distributions are ideal cases which were examined for the sake of comparison and are less biologically motivated.

There are several possible causes that would inhibit filament motion. Physical obstacles could hinder filament transport including the crowding from other filaments or crosslinking to other filaments. Stalled velocity due to the tug-of-war caused by motor molecules of opposing types could be another possible reason. Mathematical modeling shows that there are several scenarios where the majority of filaments remain in a state where the tug-of-war is unresolved resulting in stalled filaments [15]. Filaments detaching and staying detached from the microtubule would also be stalled. Finally there are direct and indirect interactions with actin which could cause anchoring of the filaments [17]. Overall, the data suggests that many of the fluorescent filaments are in the static portion of the network or in the process of being disassociated or associated with it. Thus the majority of filaments are stationary or moving with very slow velocities. This does not preclude the possibility that many filaments which are not associated with the stationary portion of the network are also pausing for long periods of time.

Assembly and disassembly of IF occurs on a time scale of hours in neurons [28]; whereas, the FRAP experiments take place on the time scale of minutes. Yet in epithelial cells, keratin assembly/disassembly occurs at the time scale of minutes [29]. But in our experiments the profile curves remain low in the middle of the bleached region indicating that assembly is not playing an important role. Preliminary results of simulations with length changes suggest that the results presented here are robust. This is not surprising since length does not substantially affect the profile curves. Of course, in systems where filament length can change at a time scale comparable to that of active transport, our analysis is not appropriate and the profile curves will have different characteristics.

Let’s consider three possible refinements to model. First, we could allow filament transport in two directions. This would be important when considering symmetric profile curves, for example those observed in astrocytes 8 hours after wounding, when cell polarity is well established [12]. Two directional analysis would indicate whether the filament velocity has the same characteristics in both directions. When there are no non-fluorescent moving filaments, the bleached region is symmetric, and the velocities are equal in magnitude but opposite in direction no new information is gained. If there are non-fluorescent, moving filaments the profile curves will be distorted but in a symmetric manner. If the velocity distributions are the same but with different mean velocities (still in opposite directions) the moving non-fluorescent filaments could break the symmetry. Second, we could allow the filament velocity to be correlated with filament length [14]. How this correlation would alter the profile curves is hard to predict without knowing how the length and speed are related. Third, we could consider the elastic nature of IFs. The elasticity of the filaments has two possible relevant effects: length change and speed change [14]. The first should not affect the profile curves but the second could. Finally, by combining the information about filament velocities found here with models of filament transport [14, 15], it should be possible to elucidate properties of motors involved in the transport. Additionally, when the cell is stationary, while the filament network is very dynamic, there is no net change in filament density.

In future mathematical models, we plan to investigate conditions which would allow a steady state density and give insight into how the cell maintains a dynamic network with no net filament transport. Further mathematical analysis could be done using other mathematical formalization. A standard method would be to use the Chapman-Kolmogorov equation, but for our problem that requires simplification. All three types of simulations can naturally be thought of as realizations of stochastic processes. With some work and simplifying assumptions, they can be framed as *Markov* processes. Type 1 simulations, while certainly stochastic, have all of the randomness front-loaded at time 0. Consequently, they are trivially Markovian because the state of the system at each strictly positive time depends deterministically on the state at any previous time. The easiest way to frame type 2 and type 3 simulations as Markovian is to consider *only* the velocity process *and* to require the elapsed time between velocity jumps to be exponentially-distributed (rather than uniform), leaving us with classical examples of continuous-time, time-homogeneous Markov processes. For interested readers the resulting Chapman-Kolmogorov equation and other related formulas are given in S2 Appendix.

In summary, the modeling framework proposed in this work provides an in-silico platform to study the impact of IF protein post-translational modifications [30] or mutations [31], depleting or silencing one motor type, or altering the IF network composition on the IF transport and organization in cells.

## Supporting information

S1 Appendix Appendix 1. (PDF)

S2 Appendix Appendix 2. (PDF)

S1 Datasets The FRAP data. (XLSX)
