# Supplementary material for: Using Fluorescence Recovery After Photobleaching data to uncover filament dynamics
Source: PLoS Comput Biol. 2022 Sep 26;18(9):e1010573. doi: 10.1371/journal.pcbi.1010573 (PMC9536589; doi:10.1371/journal.pcbi.1010573)
Supplement: S1 Appendix — (PDF) [file pcbi.1010573.s001.pdf]

SI Appendix 1: Using Fluorescence Recovery After Photobleaching data to uncover filament dynamics

J. C. Dallon[1,*], Cécile Leduc[2,3], Christopher P. Grant[1], Emily J. Evans[1], Sandrine Etienne-Manneville[2], Stéphanie Portet[4]

1 Department of Mathematics, Brigham Young University, Provo, Utah, United States of America
2 Institut Pasteur, Université de Paris, UMR3691 CNRS, Cell Polarity, Migration and Cancer Unit, Université de Paris, Equipe Labellisée Ligue Contre le Cancer, Paris, France
3 Université Paris Cité, CNRS, Institut Jacques Monod, Paris, France
4 Department of Mathematics, University of Manitoba, Winnipeg, Manitoba, Canada

Appendix 1

Consider the first probability
$\mathbb{P}((X_0, V, L) \in U) = E(\mathbb{1}_U) = \int \mathbb{1}_U(\omega)\, dP(\omega) = \int \mathbb{1}_U(x_0, v, \ell)\, dP$, where P is the distribution of (X_0, V, L) and E is the corresponding expectation. The profile curves in FRAP are scaled version of

$$H(t, y) = \int_V \int_L \int_{X_0} (\mathbb{1}_{U(t,y)}(x_0, v, \ell) + \mathbb{1}_{B(t,y)}(x_0, v, \ell))\, d\mu_{X_0}\, d\mu_L\, d\mu_V. \tag{1}$$

The intensity curve is a scaled version of $G(t) = \int_{y_0}^{y_1} H(t, y)\, dy$. Assuming X_0 is uniformly distributed on the interval $[0, F]$, $F \geq y_0$ and $w = y - vt$, then

$$\begin{aligned}
f(t, y, \ell, v) &= \int_{X_0} (\mathbb{1}_{U(t,y)}(x_0, v, \ell) + \mathbb{1}_{B(t,y)}(x_0, v, \ell))\, d\mu_{X_0} \\
&= \int_{X_0} \mathbb{1}_{[w,\infty)\cap(-\infty,w+\ell]\cap(-\infty,y_0]}(x_0)\, d\mu_{X_0} \\
&+ \int_{X_0} \mathbb{1}_{(-\infty,y_0]}(w) \mathbb{1}_{(-\infty,w+\ell]\cap[y_0,\infty)\cap(-\infty,y_0+\ell]}(x_0)\, d\mu_{X_0} \\
&= \frac{1}{F} \left[\int_0^a \mathbb{1}_{[w,\infty)\cap(-\infty,w+\ell]\cap(-\infty,y_0]}(x_0)\, dx_0 \right. \\
&\left. + \int_0^a \mathbb{1}_{(-\infty,y_0]}(w) \mathbb{1}_{(-\infty,w+\ell]\cap[y_0,\infty)\cap(-\infty,y_0+\ell]}(x_0)\, dx_0 \right] \\
&= \frac{1}{F}(I + II).
\end{aligned}$$

We consider the two integrals separately.

I If $\ell \leq y_0$

$$\int_0^F \mathbb{1}_{[w,\infty)\cap(-\infty,w+\ell]\cap(-\infty,y_0]}(x_0)\, dx_0 = \begin{cases} 0 & \text{if } w \leq -\ell \\ w + \ell & \text{if } -\ell < w \leq 0 \\ \ell & \text{if } 0 < w \leq y_0 - \ell \\ y_0 - w & \text{if } y_0 - \ell < w \leq y_0 \\ 0 & \text{if } y_0 < w \end{cases} \tag{2}$$

else if $y_0 < \ell$

$$\int_0^F \mathbb{1}_{[w,\infty)\cap(-\infty,w+\ell]\cap(-\infty,y_0]}(x_0)\,dx_0 = \begin{cases} 0 & \text{if } w \le -\ell \\ w+\ell & \text{if } -\ell < w \le y_0-\ell \\ y_0 & \text{if } y_0-\ell < w \le 0 \\ y_0-w & \text{if } 0 < w \le y_0 \\ 0 & \text{if } y_0 < w. \end{cases} \tag{3}$$

II If $F \le y_0+\ell$

$$\int_0^F \mathbb{1}_{(-\infty,y_0]}(w)\mathbb{1}_{(-\infty,w+\ell]\cap[y_0,\infty)\cap(-\infty,y_0+\ell]}(x_0)\,dx_0 = \begin{cases} 0 & \text{if } w \le y_0-\ell \\ w+\ell-y_0 & \text{if } y_0-\ell < w \le F-\ell \\ F-y_0 & \text{if } F-\ell < w \le y_0 \\ 0 & \text{if } y_0 < w \end{cases} \tag{4}$$

else if $y_0+\ell < F$

$$\int_0^F \mathbb{1}_{(-\infty,y_0]}(w)\mathbb{1}_{(-\infty,w+\ell]\cap[y_0,\infty)\cap(-\infty,y_0+\ell]}(x_0)\,dx_0 = \begin{cases} 0 & \text{if } w \le y_0-\ell \\ w+\ell-y_0 & \text{if } y_0-\ell < w \le y_0 \\ 0 & \text{if } y_0 < w. \end{cases} \tag{5}$$

Integrating these with respect to the non-negative random variable ℓ gives

$$\int \frac{1}{F} I\,d\mu_L = \begin{cases} \frac{1}{F}\left[\int_{-w}^{y_0-w}(w+\ell)\,d\mu_L + \int_{y_0-w}^{\infty} y_0\,d\mu_L\right] & \text{if } w \le 0 \\ \frac{1}{F}\left[\int_0^{y_0-w}\ell\,d\mu_L + \int_{y_0-w}^{\infty}(y_0-w)\,d\mu_L\right] & \text{if } 0 < w \le y_0 \\ 0 & \text{if } y_0 < w. \end{cases}$$

Also

$$\int \frac{1}{F} II\,d\mu_L = \begin{cases} \frac{1}{F}\left[\int_{y_0-w}^{F-w}(w+\ell-y_0)\,d\mu_L + \int_{F-w}^{\infty}(F-y_0)\,d\mu_L\right] & \text{if } w \le y_0 \\ 0 & \text{if } y_0 < w. \end{cases}$$

Thus

$$\int f(t,y,\ell,v)\,d\mu_L = \begin{cases} \frac{1}{F}\left[\int_{-w}^{F-w}(w+\ell)\,d\mu_L + \int_{F-w}^{\infty} F\,d\mu_L\right] & \text{if } w \le 0 \\ \frac{1}{F}\left[\int_0^{F-w}\ell\,d\mu_L + \int_{F-w}^{\infty}(F-w)\,d\mu_L\right] & \text{if } 0 < w \le y_0 \\ 0 & \text{if } y_0 < w. \end{cases} \tag{6}$$

If we integrate with respect to the non-negative random variable V instead we get for $\ell \le y_0$

$$\int \frac{1}{F} I\,d\mu_V = \frac{1}{F}\left[\int_{\frac{y-y_0}{t}}^{\frac{y-y_0+\ell}{t}}(y_0-y+vt)\,d\mu_V + \int_{\frac{y-y_0+\ell}{t}}^{\frac{y}{t}}\ell\,d\mu_V + \int_{\frac{y}{t}}^{\frac{y+\ell}{t}}(y-vt+\ell)\,d\mu_V\right]$$

for $\ell > y_0$

$$\int \frac{1}{F} I\,d\mu_V = \frac{1}{F}\left[\int_{\frac{y-y_0}{t}}^{\frac{y}{t}}(y_0-y+vt)\,d\mu_V + \int_{\frac{y}{t}}^{\frac{y+\ell-y_0}{t}} y_0\,d\mu_V + \int_{\frac{y+\ell-y_0}{t}}^{\frac{y+\ell}{t}}(y-vt+\ell)\,d\mu_V\right].$$

For $F \leq y_0 + \ell$

$$\int \frac{1}{F} II \, d\mu_V = \frac{1}{F} \left[\int_{\frac{y-y_0}{t}}^{\frac{y-F+\ell}{t}} (F - y_0) \, d\mu_V + \int_{\frac{y-F+\ell}{t}}^{\frac{y+\ell-y_0}{t}} (y - vt + \ell - y_0) \, d\mu_V \right]$$

and if $F > y_0 + \ell$ then

$$\int \frac{1}{F} II \, d\mu_V = \frac{1}{F} \left[\int_{\frac{y-y_0}{t}}^{\frac{y-y_0+\ell}{t}} (y - vt + \ell - y_0) \, d\mu_V \right].$$

Thus if $\ell \leq y_0$ and $F \leq y_0 + \ell$

$$\int f(t, y, \ell, v) \, d\mu_V = \frac{1}{F} \left[\int_{\frac{y-F+\ell}{t}}^{\frac{y+\ell}{t}} \ell \, d\mu_V + \int_{\frac{y-y_0}{t}}^{\frac{y-F+\ell}{t}} (F - y + vt) \, d\mu_V + \int_{\frac{y}{t}}^{\frac{y+\ell}{t}} (y - vt) \, d\mu_V \right].$$

If $\ell \leq y_0$ and $F > y_0 + \ell$

$$\int f(t, y, \ell, v) \, d\mu_V = \frac{1}{F} \left[\int_{\frac{y-y_0}{t}}^{\frac{y+\ell}{t}} \ell \, d\mu_V + \int_{\frac{y}{t}}^{\frac{y+\ell}{t}} (y - vt) \, d\mu_V \right].$$

If $\ell > y_0$ and $F \leq y_0 + \ell$ then if $F - \ell < 0$

$$\int f(t, y, \ell, v) \, d\mu_V = \frac{1}{F} \left[\int_{\frac{y-F+\ell}{t}}^{\frac{y+\ell}{t}} (y - vt + \ell) \, d\mu_V + \int_{\frac{y-y_0}{t}}^{\frac{y-F+\ell}{t}} F \, d\mu_V + \int_{\frac{y-y_0}{t}}^{\frac{y}{t}} (vt - y) \, d\mu_V \right]$$

or $\ell > y_0$ and $F \leq y_0 + \ell$ then if $F - \ell > 0$

$$\int f(t, y, \ell, v) \, d\mu_V = \frac{1}{F} \left[\int_{\frac{y-F+\ell}{t}}^{\frac{y+\ell}{t}} (y - vt + \ell) \, d\mu_V + \int_{\frac{y-y_0}{t}}^{\frac{y-F+\ell}{t}} F \, d\mu_V + \int_{\frac{y-y_0}{t}}^{\frac{y}{t}} (vt - y) \, d\mu_V \right].$$

Finally if $\ell > y_0$ and $F > y_0 + \ell$

$$\int f(t, y, \ell, v) \, d\mu_V = \frac{1}{F} \left[\int_{\frac{y-y_0}{t}}^{\frac{y+\ell}{t}} (y - vt + \ell) \, d\mu_V + \int_{\frac{y-y_0}{t}}^{\frac{y}{t}} (vt - y) \, d\mu_V \right]. \tag{7}$$
