# Supplementary material for: Using Fluorescence Recovery After Photobleaching data to uncover filament dynamics
Source: PLoS Comput Biol. 2022 Sep 26;18(9):e1010573. doi: 10.1371/journal.pcbi.1010573 (PMC9536589; doi:10.1371/journal.pcbi.1010573)
Supplement: S2 Appendix — (PDF) [file pcbi.1010573.s002.pdf]

SI Appendix 2: Using Fluorescence Recovery After Photobleaching data to uncover filament dynamics

J. C. Dallon[1,*], Cécile Leduc[2,3], Christopher P. Grant[1], Emily J. Evans[1], Sandrine Etienne-Manneville[2], Stéphanie Portet[4]

1 Department of Mathematics, Brigham Young University, Provo, Utah, United States of America
2 Institut Pasteur, Université de Paris, UMR3691 CNRS, Cell Polarity, Migration and Cancer Unit, Université de Paris, Equipe Labellisée Ligue Contre le Cancer, Paris, France
3 Université Paris Cité, CNRS, Institut Jacques Monod, Paris, France
4 Department of Mathematics, University of Manitoba, Winnipeg, Manitoba, Canada

Appendix 2

In type 2 and 3 simulations, if we discard the position process, retaining only the velocity process $V(t)$, *and* if we assume that velocities are valid for exponentially distributed durations then we have classical examples of time-homogeneous Markov processes. Additionally for type 3 simulations we will assume that there are only two velocity: 0 and a non-negative speed s.

One way Markov processes are commonly analyzed is using the Chapman-Kolmogorov equation. Let the transition kernel, K be defined as

$$K(t,v,\Gamma) := \mathbb{P}(V(t) \in \Gamma \mid V(0) = v);$$

then the Chapman-Kolmogorov equation is

$$K(t+s,v,\Gamma) = \int K(s,y,\Gamma)K(t,v,dy).$$

For type 2 simulations K is

$$K(t,v,\Gamma) = \mathbb{1}_\Gamma(v)\exp(-t/\tau_{\text{on}}) + \mu_V(\Gamma)(1-\exp(-t/\tau_{\text{on}})).$$

For type 3 K is

$$\begin{aligned}K(t,v,\Gamma) = \frac{1}{\tau_{\text{on}}+\tau_{\text{off}}}\{&\mathbb{1}_\Gamma(0)[\mathbb{1}_{\{0\}}(v)(\tau_{\text{off}}+\tau_{\text{on}}g(t)) + \tau_{\text{off}}\mathbb{1}_{\{s\}}(v)(1-g(t))]\\ &+\mathbb{1}_\Gamma(s)[\mathbb{1}_{\{s\}}(v)(\tau_{\text{on}}+\tau_{\text{off}}g(t)) + \tau_{\text{on}}\mathbb{1}_{\{0\}}(v)(1-g(t))]\}\end{aligned}$$

where $g(t) = \exp(-\frac{t}{\tau_{\text{on}}} - \frac{t}{\tau_{\text{off}}})$, (see, *e.g.*, [1]). Then K can be simplified to

$$\begin{aligned}&K(t,v,\Gamma) = \mathbb{1}_\Gamma(0)\\ &+(\mathbb{1}_\Gamma(s)-\mathbb{1}_\Gamma(0))\left[\left(1-\exp\left(-\frac{t}{\tau_{\text{on}}}-\frac{t}{\tau_{\text{off}}}\right)\right)\left(\frac{\tau_{\text{on}}}{\tau_{\text{on}}+\tau_{\text{off}}}-\mathbb{1}_{\{s\}}(v)\right)+\mathbb{1}_{\{s\}}(v)\right].\end{aligned}$$

References

1. Liggett TM. Continuous time Markov processes. vol. 113 of Graduate Studies in Mathematics. American Mathematical Society, Providence, RI; 2010. Available from: `https://doi.org/10.1090/gsm/113`.
